# Toward Efficient Two‐Photon Circularly Polarized Light Detection through Cooperative Strategies in Chiral Quasi‐2D Perovskites

**DOI:** 10.1002/advs.202206070

**Published:** 2023-01-22

**Authors:** Wentao Wu, Xiaoying Shang, Zhijin Xu, Huang Ye, Yunpeng Yao, Xueyuan Chen, Maochun Hong, Junhua Luo, Lina Li

**Affiliations:** ^1^ State Key Laboratory of Structural Chemistry Fujian Institute of Research on the Structure of Matter Chinese Academy of Sciences Fuzhou Fujian 350002 P. R. China; ^2^ University of Chinese Academy of Sciences Beijing 100049 P. R. China; ^3^ CAS Key Laboratory of Design and Assembly of Functional Nanostructures and Fujian Key Laboratory of Nanomaterials Fujian Institute of Research on the Structure of Matter Chinese Academy of Sciences Fuzhou Fujian 350002 P. R. China; ^4^ Fujian Science and Technology Innovation Laboratory for Optoelectronic Information of China Fuzhou Fujian 350108 P. R. China; ^5^ School of Chemistry and Chemical Engineering Jiangxi Normal University Nanchang Jiangxi 330022 P. R. China

**Keywords:** chemical design, chiral multilayered perovskites, circularly polarized light detection, near‐infrared, two‐photon absorption

## Abstract

Organic–inorganic hybrid perovskites carry unique semiconducting properties and advanced flexible crystal structures. These characteristics of organic–inorganic hybrid perovskites create a promising candidacy for circularly polarized light (CPL) detection. However, CPL detections based on chiral perovskites are limited to UV and visible wavelengths. The natural quantum well structures of layered hybrid perovskites generate strong light–matter interactions. This makes it possible to achieve near‐infrared (NIR) CPL detection via two‐photon absorption in the sub‐wavelength region. In this study, cooperative strategies of dimension increase and mixed spacer cations are used to obtain a pair of chiral multilayered perovskites (R‐*β*‐MPA)EA_2_Pb_2_Br_7_ and (S‐*β*‐MPA)EA_2_Pb_2_Br_7_ (MPA = methylphenethylammonium and EA = ethylammonium). The distinctive bi‐cations interlayer and multilayered inorganic skeletons provide enhanced photoconduction. Moreover, superior photoconduction leads to the prominent NIR CPL response with a responsivity up to 8.1 × 10^−5^ A W^−1^. It is anticipated that this work can serve as a benchmark for the fabrication and optimization of efficient NIR CPL detection by simple chemical design.

## Graphical abstract

Two chiral multilayered perovskites with alternating cations in the interlayer are unprecedentedly obtained by dimension increase and mixed spacer cations. The distinctive structure gives rise to an enhancement of photoconduction, leading to the high near‐infrared (NIR) circularly polarized light (CPL) responsivity (8.1 × 10^−5^ A W^−1^). This work highlights the design flexibility of hybrid perovskites for high‐performance NIR CPL detection.

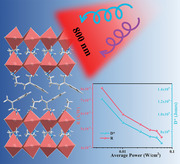

## 1 Introduction

Organic–inorganic hybrid perovskites have been widely used in for photoelectronic applications because of their excellent photoelectronic properties. These properties include low defect density,^[^ ^1^ ^]^ long carrier diffusion,^[^ ^2^, ^3^, ^4^, ^5^ ^]^ and high optical absorption coefficient.^[^ ^6^ ^]^ It is worth noting that the structural flexibility of low‐dimensional perovskites allows chiral organic cations to be inserted into perovskite layers to endow chirality.^[^ ^7^ ^]^ The chiral optical activity of perovskites can be realized through the transfer of chirality from the organic cations to the inorganic skeletons.^[^ ^8^ ^]^ Therefore, the direct detection of circularly polarized light (CPL) using 2D chiral perovskites has attracted great attention in the fields of chiroptoelectronics.^[^ ^9^, ^10^, ^11^, ^12^, ^13^, ^14^, ^15^ ^]^ Tang et al. realized high‐performance CPL detection employing chiral hybrid perovskites, (R‐/S‐*α*‐MBA)PbI_4_ (MBA = phenylethylamine).^[^ ^9^ ^]^ Wu group using a 2D chiral perovskite nanowires achieved Stokes photodetection with impressive detectivity.^[^ ^11^ ^]^ Despite those significant achievements, most of the CPL photodetections based on chiral perovskites can only work in UV and visible wavelengths. The main reason is that the typical chiral 2D perovskites exhibit chiral optical inactivity in the near‐infrared (NIR) region limited by their optical bandgap.

As a nonlinear optical process, two‐photon absorption can convert light with photon energies below the bandgap into electrical signals and has been widely used in sub‐bandgap photodetection.^[^ ^16^ ^]^ The natural quantum well structure in 2D hybrid perovskites can generate strong light–matter interactions, which makes it possible to obtain high optical nonlinearity in the sub‐bandgap region.^[^ ^17^, ^18^, ^19^, ^20^, ^21^, ^22^ ^]^ Thus, chiral 2D hybrid perovskites are expected to realize NIR circularly polarized photoelectric detection via two‐photon absorption. In this context, our group has achieved vis−NIR dual‐modal CPL detection base on a chiral Ruddlesden–Popper perovskite, (*R*‐BPEA)_2_PbI_4_ (*R*‐BPEA = (*R*)‐1‐(4‐ bromophenyl)ethylammonium).^[^ ^23^ ^]^ Nevertheless, this chiral perovskite showed relatively lower responsivity (3.5 × 10^−6^ A W^−1^) for NIR CPL detection. Therefore, how to acquire high‐performance CPL response of 2D hybrid perovskites in sub‐bandgap deserves our intense efforts.

In this study, dimension increase and mixed spacer cations were applied and yielded newly synthesized two chiral multilayered perovskites with alternating cations in the interlayer (ACI perovskites), (*R*‐*β*‐MPA)EA_2_Pb_2_Br_7_ (**R‐2**) and (S‐*β*‐MPA)EA_2_Pb_2_Br_7_ (**S‐2**, MPA = methylphenethylammonium and EA = ethylammonium), The unique multilayered ACI structure enables **R/S‐2** superior semiconducting properties without the losses of chirality, including narrower band gap, higher photoresponse, and faster response speed. Under the circumstances, high‐sensitive NIR CPL detection with a responsivity up to 8.1 × 10^−5^ A W^−1^ was achieved for **R‐2**.

## 2 Results and Discussion

### 2.1 Structure Analysis

**Figure 1:**
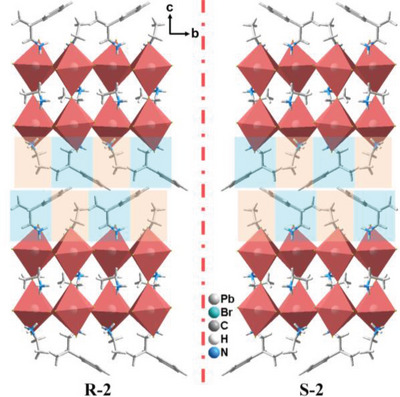
Enantiomeric structures of **R‐2** and **S‐2** viewed along *a* axis.

**R‐2** and **S‐2** are antisymmetrically isostructural and crystallize in the chiral space group of *P*2_1_ assigned to the monoclinic crystal system (Table S1, Supporting Information). The single‐crystal structures of them are characteristic of quasi‐2D perovskites with multilayered [Pb_2_Br_7_]^3−^ inorganic skeleton at room temperature (**Figure** **1**). The inorganic bilayers are composed of corner‐sharing [PbBr_6_]^4−^ octahedra. As shown in Figure 1, EA^+^ plays the both roles of “perovskitizer” cations and “spacer” cations. However, EA^+^ only takes half the position of organic layers and the other half is occupied by chiral *R* or *S*‐*β*‐MPA^+^ (*R*/*S*‐*β*‐MPA^+^). As a result, the alternating EA^+^ (yellow background) and *R*/*S*‐*β*‐MPA^+^ (blue background) form the distinct ACI perovskites. Moreover, the asymmetric hydrogen bonding interaction between *R*/*S*‐*β*‐MPA^+^ and inorganic octahedra contributes to the chiroptical activity of **R‐2** and **S‐2** (Figure S1, Supporting Information).^[^ ^24^ ^]^ In this way, we successfully achieved multilayered ACI perovskites with the maintenance of chirality.

**Figure 2:**
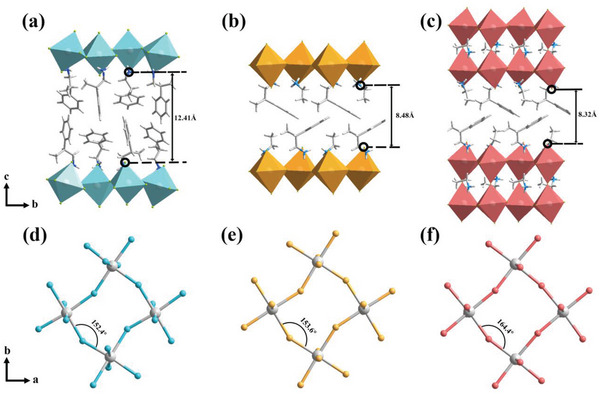
Barrier thickness (minimal distances between terminal bromine ions) of a) **R‐0**, b) **R‐1**, and c) **R‐2**. Pb−Br−Pb angles of d) **R‐0**, e) **R‐1**, and f) **R‐2**.

Before studying the photoelectric performance of these multilayered ACI perovskites, the structural differences among (*R*‐*β*‐MPA)_2_PbBr_4_^[^ ^13^ ^]^ (**R‐0**), (*R*‐*β*‐MPA)EAPbBr_4_^[^ ^25^ ^]^ (**R‐1**), and **R‐2** were investigated. The single‐crystal structures of **R‐0**, **R‐1**, and **R‐2** were collected at room temperature (≈300 K). The crystal data and structure refinements are shown in Table S2 in the Supporting Information. From **R‐0** to **R‐2**, the introduction of EA shrinks the volume of “spacer” cations and reduces the barrier thickness (**Figure** **2**a–c), leading to stronger coupling of the Br p‐orbitals and reduced dielectric mismatch of the interlayer, which may further contribute to the decrease of the band gap and exciton binding energy.^[^ ^26^, ^27^ ^]^ Thus, the narrower band gap and lower exciton binding energy facilitate the dissociating of excitons and enhance the photocurrent. In addition, as the distortion level of the inorganic octahedra is also closely related to the band gap and localization of electron–hole pairs of the materials,^[^ ^28^, ^29^ ^]^ the distortion of **R‐0**, **R‐1**, and **R‐2** was investigated. As exhibited in Figure 2d–f, **R‐2** presents the biggest average Pb−Br−Pb angles of 164.4°, corresponding to the smallest lattice distortion. In contrast, **R‐0** has the most distorted lattice with Pb−Br−Pb angles of 152.4°. Since the photoluminescence (PL) properties of 2D hybrid perovskites are sensitive to the distortions of the inorganic skeleton,^[^ ^30^, ^31^, ^32^ ^]^ narrower full‐width at half‐maximum of PL spectra (Figure S2, Supporting Information) and shorter PL lifetime (Figure S3, Supporting Information) of **R‐2** at room temperature further confirm the distortion reductions,^[^ ^32^ ^]^ which potentially contributes to the enhancement of photoconduction.

### 2.2 Chiral and Semiconducting Properties

**Figure 3:**
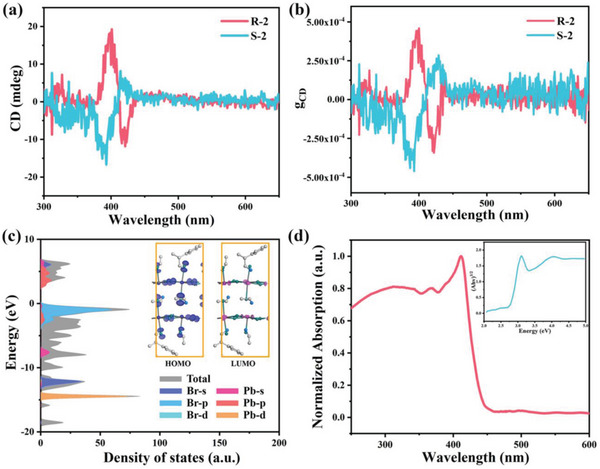
a) CD and b) *g*_CD_ spectra of **R‐2** and **S‐2**. c) The DOS and charge density distributions of the relevant orbitals of **R‐2**. d) Linear absorption spectra of **R‐2** and the deduced optical bandgap.

The powder samples of (*R*/*S*‐*β*‐MPA)EA_2_Pb_2_Br_7_ were pulverized by their single‐crystals. Powder X‐ray diffraction (PXRD) verified their purity (Figure S4, Supporting Information). As shown in **Figure** **3**a, **R‐2** and **S‐2** show opposite circular dichroism (CD) signals around exciton absorption (Figure S5, Supporting Information), verifying the transfer of chirality from *R/S*‐*β*‐MPA to the inorganic layers. In addition, bisignate CD signals can be observed from one configuration with two peaks at 397 and 422 nm, known as Cotton effect, which is attributed to the splitting of the energy state with the opposite spin states of electrons.^[^ ^33^, ^34^, ^35^ ^]^ The anisotropy factor *g*_CD_ (Figure 3b) was calculated based on the following equation^[^ ^25^ ^]^

(1) $$g_{\mathrm{CD}}=\frac{\mathrm{CD}[\mathrm{mdeg}]}{32980\times \mathrm{Absorbance}}$$

the maximum of *g*_CD_ locates at 393 nm with a value of 4.6 × 10^−4^, which is comparable to those of some reported 2D chiral perovskites.^[^ ^13^, ^36^, ^37^ ^]^

Density of states (DOS) and charge density distributions (Figure 3c) reveal that the Pb 6p and Br 4p contribute the most to the conduction band minimum and the valence band maximum, respectively, indicating the crucial role of inorganic skeletons in determining the semiconducting properties of **R‐2**, which theoretically verify the reasonability of our strategies. The linear absorption spectra were performed on **R/S‐2** (Figure 3d and Figure S5, Supporting Information), showing a cutoff of 451 nm and corresponding to an optical band gap of 2.75 eV (Figure 3d inset). Meanwhile, compared with those of **R‐0** and **R‐1**, the narrower band gap of **R‐2** (Figure S6, Supporting Information) is in agreement with our speculation from the single‐crystal structures, and improved photoresponse is promising for **R‐2**.

### 2.3 Photoelectric Response

**Figure 4:**
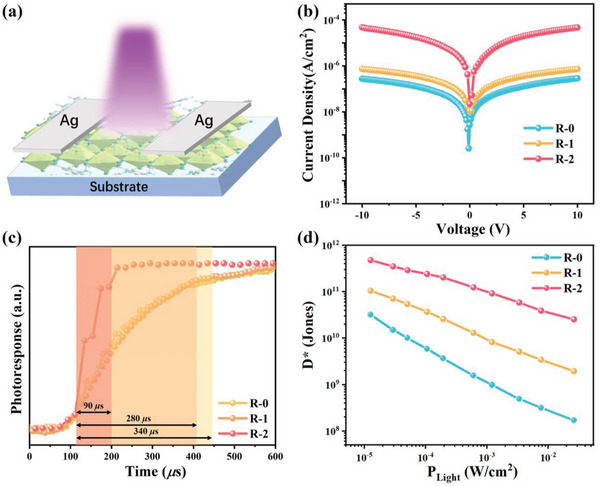
a) Photodetection device illustration. b) *J–V* curves under 405 nm laser with the incident light intensity of 26.3 mW cm^−2^. c) Rise of photoresponse of **R‐0**, **R‐1**, and **R‐2**. d) Light intensity‐dependent detectivity of **R‐0**, **R‐1**, and **R‐2**.

Single‐crystal‐based photodetectors of **R‐0**, **R‐1**, and **R‐2** were fabricated on the (001) plane (parallel to the inorganic layer) to verify the photoresponse improvement from **R‐0** to **R‐2**. Device illustration is shown in **Figure** **4**a. Under 405 nm (26.3 mW cm^−2^) irradiation, all three samples exhibited obvious photoresponse and the current density showed a dramatic increase from **R‐0** to **R‐2** (Figure 4b). The on/off current ratio of **R‐2** is about 3300, two orders of magnitude higher than that of **R‐0**. The rise (*τ*_rise_) and fall (*τ*_fall_) time are defined as the time to increase or reduce the photoresponse from 10% to 90%. **R‐2** showed a marvelously fast response speed with both *τ*_rise_ and *τ*_fall_ less than 100 µs (Figure 4c and Figure S7, Supporting Information), which were only about 30% of that of **R‐1** and faster than most of reported 2D hybrid perovskite single‐crystal‐based photodetection.^[^ ^38^, ^39^, ^40^, ^41^ ^]^ The detectivity (D*) of **R‐0**, **R‐1**, and **R‐2** was further calculated according to the equation D* = *I*_ph_/[*P*_i_×(2*eI*_d_)^1/2^], where *I*_ph_ is the photocurrent, *P*_i_ is the incident power, and *I*_d_ is the dark current. As shown in Figure 4d, the maximum detectivity of **R‐2** reached 4.8 × 10^11^ Jones, which is not only much higher than that of **R‐0**, but also better than many other quasi‐2D hybrid perovskites photodetectors.^[^ ^38^, ^39^, ^41^ ^]^ All these results verify that our strategies successfully improved the photoresponse of our hybrid perovskite (**R‐2**) and imply its great potential in efficient NIR CPL photodetection.

### 2.4 Sub‐Bandgap CPL Photodetection

**Figure 5:**
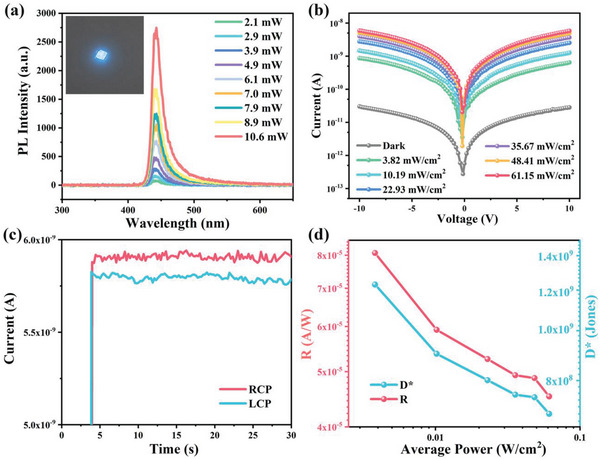
a) Excitation intensity‐dependent emission spectra of **R‐2** at 800 nm. b) The photoresponse of **R‐2** under 800 nm laser. c) The current–time curves under 800 nm CPL. d) Light intensity‐dependent detectivity and responsivity of **R‐2** at 10 V bias.

Before the investigation of NIR CPL photoresponse of **R‐2**, we studied its two‐photon response ability. As shown in **Figure** **5**a, R‐2 showed a strong blue‐light emission with the peak intensity located at 443 nm under 800 nm excitation, same to that excited by 365 nm light. Moreover, the PL intensity exhibited obvious excitation intensity dependence and the logarithmic power‐dependent PL peak intensity showed a slope of 2.11 (Figure S8, Supporting Information), which is the smoking gun of the two‐photon response in **R‐2**. The photoelectric response of **R‐2** under 800 nm pump laser was measured with different incident intensity (Figure 5b). As the light intensity increased, the photocurrent increased dramatically. The photocurrent (*V*_bias_ = 10 V) under 61.15 mW cm^−2^ was more than 200 times larger than that in the dark. Figure 5c shows the NIR CPL response of **R‐2**, the photocurrent varied depending on whether it was excited by the right‐handed CPL (RCP) or left‐handed CPL (LCP), although the intensities of the light were the same during the measurements. **R‐2** showed an obviously higher response under RCP light. The calculated photocurrent difference *g_I_*_ph_ (*g_I_*_ph_ = 2(*I*_R_ − *I*_L_)/(*I*_R_ + *I*_L_), where *I*_R_ and *I*_L_ are the photocurrents under RCP and LCP irradiation) is about 0.03. Relatively lower *g_I_*_ph_ might be associated with a smaller percentage of chiral organic ligands.^[^ ^42^ ^]^ The light intensity‐dependent responsivity (*R*, *R* = *I*_ph_/*P*_i_) and detectivity (D*) were further investigated (Figure 5d and Figure S9, Supporting Information). The highest D* reached 1.2 × 10^9^ Jones, which is even higher than that of some interband CPL photodetections.^[^ ^41^ ^]^ The calculated highest *R* was about 8.1 × 10^−5^ A W^−1^. As far as we know, such responsivity is higher than most of the reported hybrid perovskite‐based two‐photon photodetectors, including CH_3_NH_3_PbBr_3_ (≈10^−7^ A W^−1^ at 800 nm),^[^ ^43^ ^]^ CH_3_NH_3_Pb_0.75_Sn_0.25_I_3_ (≈10^−8^ A W^−1^ at 1535 nm),^[^ ^44^ ^]^ (Br‐BA)_2_(EA)_2_Pb_3_Br_10_ (10^−7^ A W^−1^ at 800 nm),^[^ ^45^ ^]^ especially, is more than one order of magnitude higher than that of the NIR CPL photodetector (R‐BPEA)_2_PbI_4_ (3.5 × 10^−6^ A W^−1^ at 800 nm).^[^ ^23^ ^]^ All these results confirm the effectiveness of our strategies in improving sub‐bandgap CPL photoresponse.

## 3 Conclusion

We presented two chiral quasi‐2D ACI perovskites (R‐*β*‐MPA)EA_2_Pb_2_Br_7_ and (S‐*β*‐MPA)EA_2_Pb_2_Br_7_ for the first time by using the cooperative strategies of dimension increase and mixed spacer cations. The inorganic skeletons in these perovskites with increased layer number and reduced distortion give rise to the notable improvement of photoresponse. Strikingly, benefitting from the improved photoconduction, (R‐*β*‐MPA)EA_2_Pb_2_Br_7_ achieves highly sensitive NIR CPL detection with a marvelous two‐photon responsivity of 8.1 × 10^−5^ A W^−1^. These results highlight great flexibility for the design of new chiral hybrid perovskites for high‐performance NIR CPL detection and will broaden potential optoelectronic applications of hybrid perovskites.

## 4 Experimental Section

### Materials

For the synthesis of (*R*/*S*‐*β*‐MPA)_2_PbBr_4_, 0.135 g (*R*)/(*S*)‐*β*‐methylphenethylammonium (1 mmol), and 0.379 g Pb(Ac)_2_·3H_2_O (1 mmol) were dissolved in 40% w/w aqueous HBr solution (5 mL) at room temperature and heated under stirring to give a clear solution. Hot solution then was cooled to room temperature to crystalize. To get the single crystals of (*R*/*S*‐*β*‐MPA)_2_PbBr_4_, the saturated solutions were prepared at 333 K and cooled down with a rate of 1 K day^−1^.

The preparation steps of (*R*/*S*‐*β*‐MPA)EAPbBr_4_ microcrystals and bulk crystals were reported in previous work.^[^ ^25^ ^]^

By increasing the proportions of EA and Pb(Ac)_2_·3H_2_O to 0.258 g (4 mmol) and 0.758 g (2 mmol), respectively, the synthesis method of (*R*/*S*‐*β*‐MPA)EA_2_Pb_2_Br_7_ was gotten. The saturated solutions were prepared at 348 K and cooled down with a rate of 1 K day^−1^ to get single crystals. It is worth noting that the as‐synthesized microcrystals of (*R*/*S*‐*β*‐MPA)EA_2_Pb_2_Br_7_ were not pure.

All reagents and solvents in the syntheses were purchased from Aladdin and used without further purification.

### Single‐Crystal Structure Determination

Single‐crystal XRDs for **R‐1**, **R‐2**, and **S‐2** were performed on a BRUKER APEX‐II diffractometer with the Mo K*α* radiation at 300 K. The APX3 software was used for data reduction. The structures were solved by direct methods and then refined by the full‐matrix least‐squares refinements on F2 using SHELXLTL software package. All the nonhydrogen atoms were located from the trial structure and refined anisotropically with SHELXTL using the full‐matrix least‐squares procedure. The structure of ***R*‐0** was quoted from another work.^[^ ^13^ ^]^

### CD and Absorption Spectra Measurement

The CD and absorption spectra of **R‐2** and **S‐2** were measured on a BioLogic MOS 450 CD spectrometer. 4 mg **R‐2** (**S‐2**) mixed with 100 mg dry pure KBr (SP grade) was ground into fine powder in agate mortar and grinded to complete mixing. Then 15 mg mixture was put in a clean mold and pressed into transparent wafer, which was placed in the beam path. The spectra were collected in the range of 250–600 nm with the scanning rate of 500 nm min^−1^, and the data interval was 1 nm.

### PL Spectra and Lifetime Measurement

The fluorescence measurements were performed on an Edinbergh Analytical instrument FLS920 at room temperature. The PL spectra of **R‐0**, **R‐1**, and **R‐2** were excited by 365 nm laser. The PL lifetime of **R‐1** and **R‐2** was collected at their PL peak and **R‐0** was collected at 424 nm. The two‐photon PL spectra of **R‐2** were excited by 800 nm femtosecond laser, which was generated by a regeneratively amplified femtosecond Ti:sapphire laser system (800 nm, 1 kHz, pulse energy of 4 mJ, pulse width of 120 fs, Spitfire Pro‐FIKXP, Spectra‐Physics).

### Calculation Method

The band gap of **R‐0**, **R‐1**, and **R‐2** and DOS of **R‐2** were performed by the density functional theory method within the total‐energy code CASTEP. The calculation details were the same with that of previous work.^[^ ^1^ ^]^

### PXRD

PXRDs were performed on a Rigaku MiniFlex 600 diffractometer equipped with a Cu K*α* radiation at room temperature from 5° to 40° with a step size of 0.2° min^−1^.

### Photoelectric Performance Measurement

All the photodetectors were fabricated on the (001) surface of the single crystals. The 405 nm laser was generated from light‐emitting diodes (THORLABS, ITC4001). CPL was generated by employing a linear polarizer (THORLABS, CRM1L) and quarter‐wave plate (THORLABS, WPQ10ME‐405). The 800 nm laser was generated by the femtosecond laser system. CPL was obtained by a linear polarizer (Thorlabs) and a quarter‐wave plate (Thorlabs). The current signals were collected using a Keithel 6517B electrometer.

CCDC 2181083, 2181070, and 2181138 contains the supplementary crystallographic data for this paper. These data can be obtained free of charge from The Cambridge Crystallographic Data Centre via www.ccdc.cam.ac.uk/data_request/cif.

## Conflict of Interest

The authors declare no conflict of interest.

## Supporting information

Supporting information

Supporting information

## Data Availability

The data that support the findings of this study are available from the corresponding author upon reasonable request.
