# Supplementary material for: Toward Efficient Two‐Photon Circularly Polarized Light Detection through Cooperative Strategies in Chiral Quasi‐2D Perovskites
Source: Adv Sci (Weinh). 2023 Jan 22;10(9):2206070. doi: 10.1002/advs.202206070 (PMC10037957; doi:10.1002/advs.202206070)
Supplement: Supplementary file 1 — Supporting information [file ADVS-10-2206070-s001.pdf]

Supporting Information

Toward Efficient Two-Photon Circularly Polarized Light Detection through Cooperative Strategies in Chiral Quasi 2D Perovskites

Wentao Wu,[1] Xiaoying Shang,[1, 3] Zhijin Xu,[1] Huang Ye,[1, 2] Yunpeng Yao,[1] Xueyuan Chen,[2, 3] Maochun Hong,[1, 2, 4] Junhua Luo,[1, 2, 4, 5] and Lina Li[1, 2, 4]*

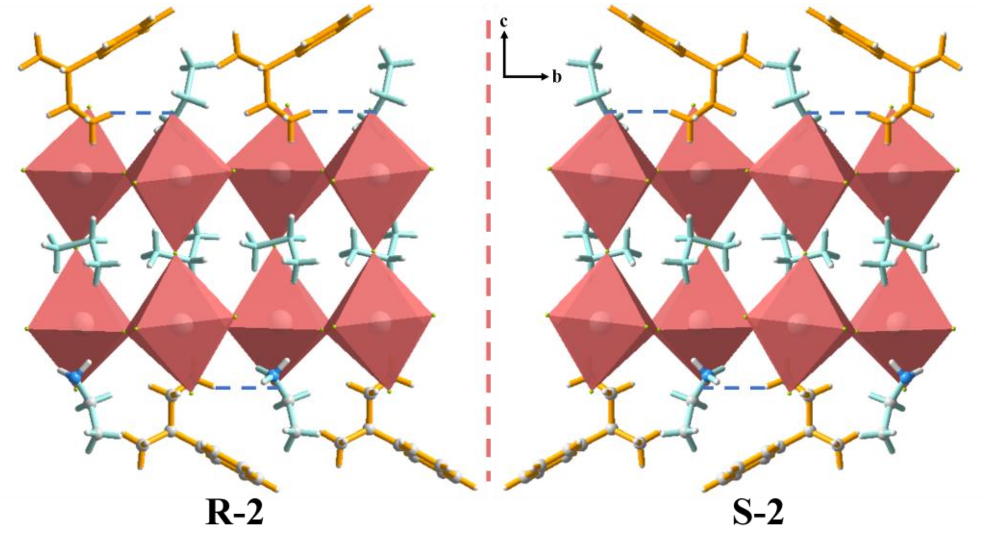

Figure S1. Schematic illustration of intra-octahedron distortions and hydrogen bondings of **R-2** and **S-2**.

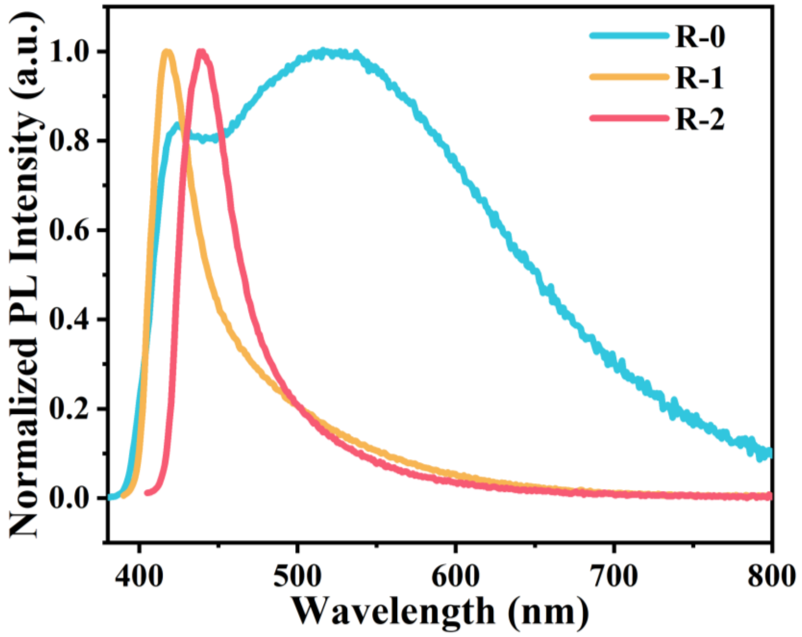

Figure S2. Normalized PL spectra of **R-0, R-1** and **R-2** powder at room temperature.

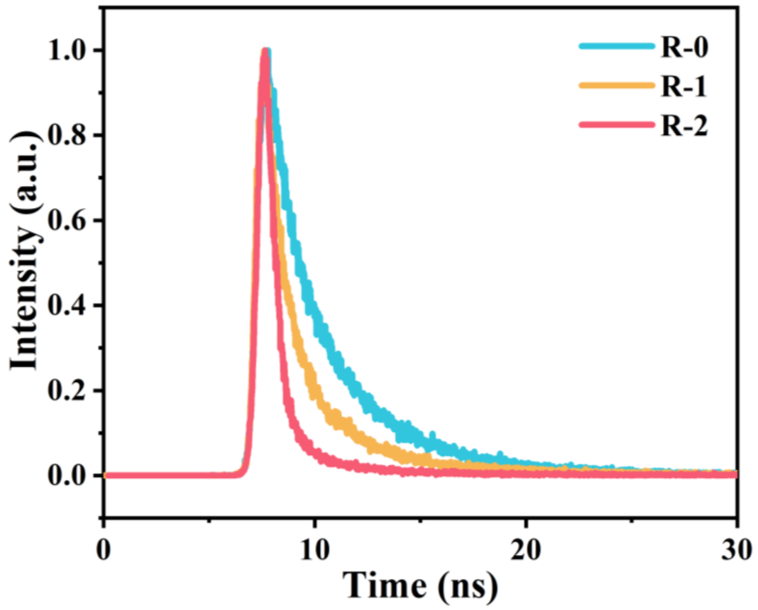

Figure S3. Fitted normalized PL lifetimes of **R-0**, **R-1** and **R-2** powder at room temperature.

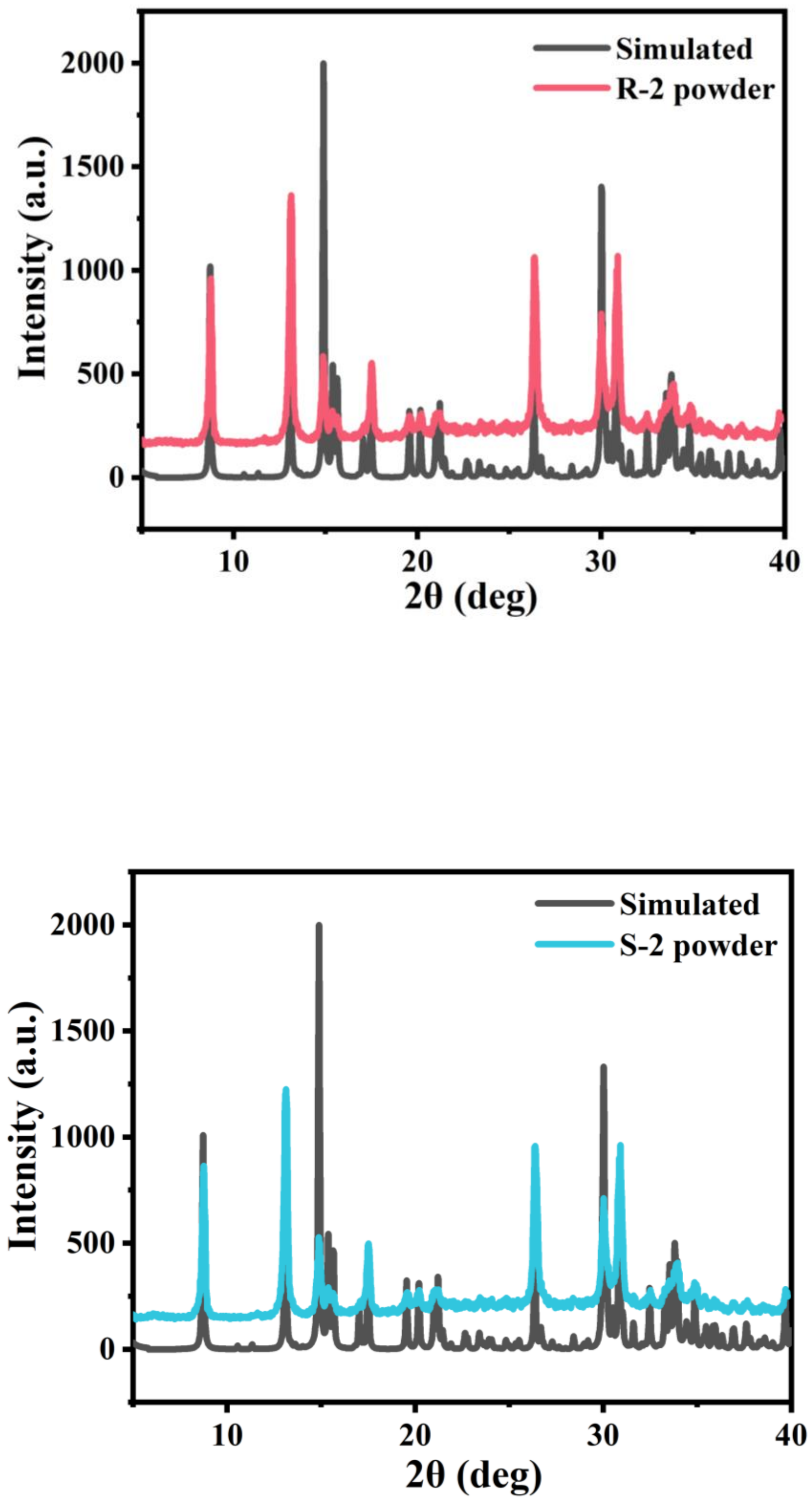

Figure S4. Simulated and experimental X-ray diffraction patterns of **R-2** and **S-2**.

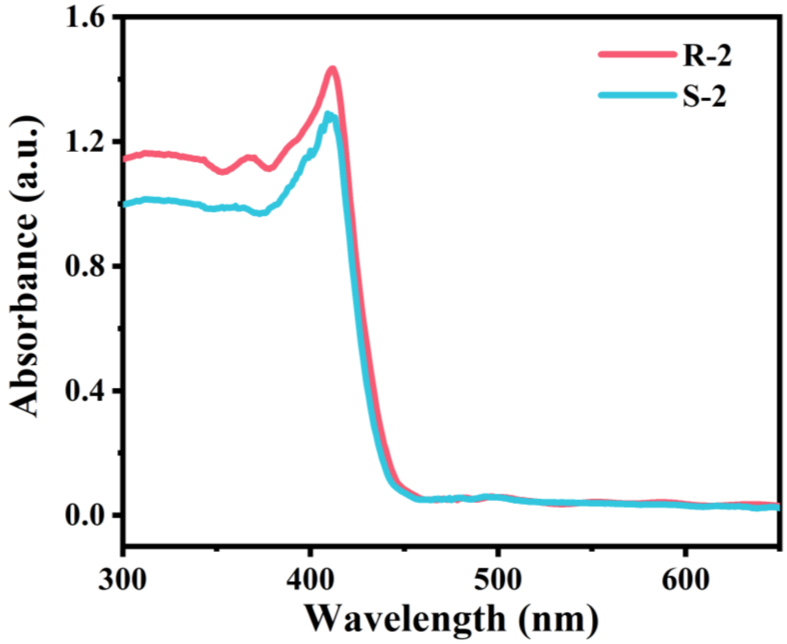

Figure S5. Absorbance of **R-2** and **S-2**.

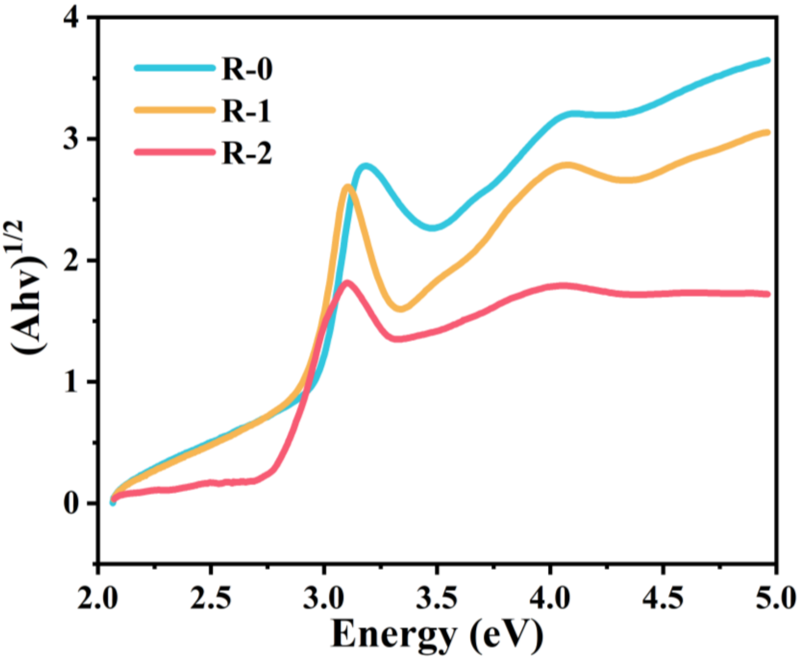

Figure S6. Calculated optical band gap of **R-0, R-1** and **R-2**.

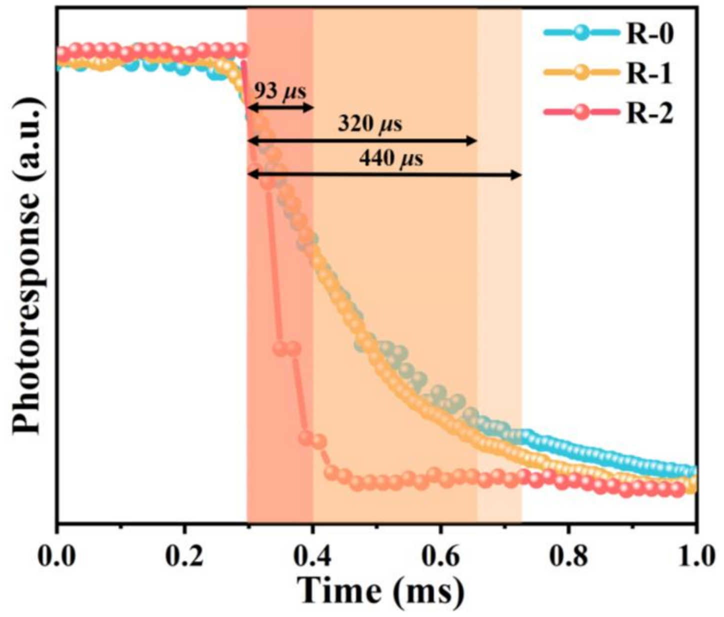

Figure S7. Fall of photoresponse of **R-0**, **R-1** and **R-2**.

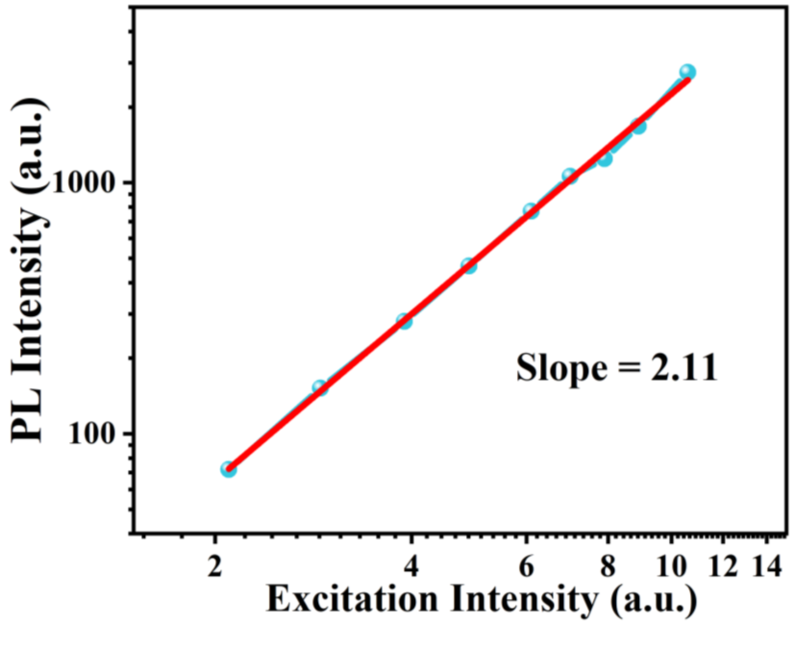

Figure S8. Plot of power-dependent PL intensity upon excitation at 800 nm for **R-2**.

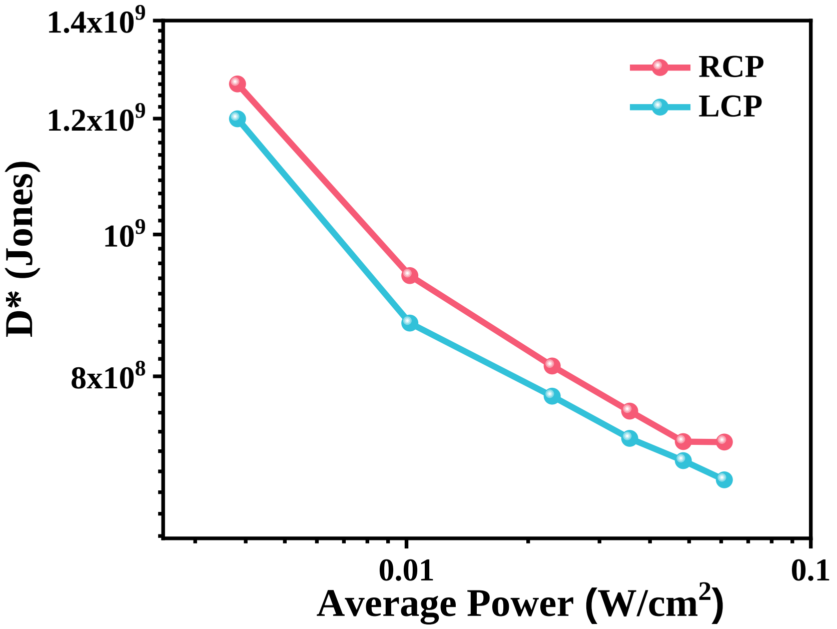

Figure S9. The detectivity of R-2-based device under LCP and RCP light at 800 nm.

Table S1. Crystal data and structure refinement for **R-2** and **S-2**.

Identification code	**R-2**	**S-2**
Empirical formula	$C_{13}H_{30}Br_7N_3Pb_2$	$C_{13}H_{30}Br_7N_3Pb_2$
Formula weight	1202.15	1202.15
Temperature/K	299.89	300.04
Crystal system	monoclinic	monoclinic
Space group	$P2_1$	$P2_1$
a/Å	8.3738(13)	8.3804(8)
b/Å	8.4518(12)	8.4545(7)
c/Å	20.304(3)	20.298(2)
α/°	90	90
β/°	92.408(5)	92.652(4)
γ/°	90	90
Volume/Å^3	1435.7(4)	1436.6(2)
Z	2	2
ρ_{calc}g/cm^3	2.781	2.779
μ/mm^{-1}	21.457	21.443
F(000)	1076.0	1076.0
Radiation	MoKα (λ = 0.71073)	MoKα (λ = 0.71073)
2θ range for data collection/°	4.868 to 55.186	5.178 to 55.27

Index ranges	$-10 \le h \le 10$, $-10 \le k \le 10$, $-24 \le l \le 26$	$-10 \le h \le 10$, $-10 \le k \le 11$, $-21 \le l \le 26$
Reflections collected	18550	19929
Independent reflections	6541 [R_{int} = 0.0853, R_{sigma} = 0.0995]	6594 [R_{int} = 0.0766, R_{sigma} = 0.0874]
Data/restraints/parameters	6541/9/220	6594/5/220
Goodness-of-fit on F^2	1.008	0.978
Final R indexes [I>=2σ (I)]	R_1 = 0.0533, wR_2 = 0.1152	R_1 = 0.0504, wR_2 = 0.1103
Final R indexes [all data]	R_1 = 0.1119, wR_2 = 0.1414	R_1 = 0.0770, wR_2 = 0.1251
Largest diff. peak/hole / e $Å^{-3}$	1.68/-2.16	2.42/-2.79
Flack parameter	0.039(17)	0.032(16)
CCDC number	2181070	2181138

Table S2. Crystal data and structure refinement for **R-0**, **R-1** and **R-2**.

Identification code	**R-0**	**R-1**	**R-2**
Empirical formula	$C_{18}H_{28}Br_4N_2Pb$	$C_{11}H_{22}Br_4N_2Pb$	$C_{13}H_{30}Br_7N_3Pb_2$
Formula weight	799.25	709.13	1202.15
Temperature/K	297.49(10)	300.02	299.89
Crystal system	orthorhombic	monoclinic	monoclinic
Space group	$P2_12_12_1$	$P2_1$	$P2_1$
a/Å	8.1348(2)	8.1695(5)	8.3738(13)
b/Å	33.7948(12)	8.3528(6)	8.4518(12)
c/Å	36.4181(11)	14.4381(13)	20.304(3)
α/°	90	90	90
β/°	90	93.110(3)	92.408(5)
γ/°	90	90	90
Volume/$Å^3$	10011.8(5)	983.78(13)	1435.7(4)
Z	16	2	2
ρ_{calc}g/cm^3	2.121	2.394	2.781
μ/mm^{-1}	13.131	16.687	21.457
F(000)	5952.0	648.0	1076.0
Radiation	MoKα (λ = 0.71073)	MoKα (λ = 0.71073)	MoKα (λ = 0.71073)

2θ range for data collection/°	3.288 to 61.794	4.994 to 55.086	4.868 to 55.186
Index ranges	$-10 \le h \le 9$, $-42 \le k \le 40$, $-45 \le l \le 44$	$-10 \le h \le 10$, $-10 \le k \le 10$, $-15 \le l \le 18$	$-10 \le h \le 10$, $-10 \le k \le 10$, $-24 \le l \le 26$
Reflections collected	63471	12860	18550
Independent reflections	22955 [R_{int}= 0.0367, R_{sigma}= 0.0501]	4508 [R_{int}= 0.0461, R_{sigma}= 0.0560]	6541 [R_{int}= 0.0853, R_{sigma}= 0.0995]
Data/restraints/parameters	22955/0/617	4508/1/155	6541/9/220
Goodness-of-fit on F^2	1.032	0.986	1.008
Final R indexes [I>=2σ (I)]	R_1= 0.0398, wR_2= 0.0775	R_1= 0.0326, wR_2= 0.0520	R_1= 0.0533, wR_2= 0.1152
Final R indexes [all data]	R_1= 0.0765, wR_2= 0.0861	R_1= 0.0455, wR_2= 0.0566	R_1= 0.1119, wR_2= 0.1414
Largest diff. peak/hole / e $Å^{-3}$	1.06/-0.90	0.68/-0.97	1.68/-2.16
Flack parameter	0.015(6)	0.018(13)	0.039(17)
CCDC number	2093821	2181083	2181070
